# Long-term clinical outcome in nasopharyngeal carcinoma patients with post-radiation persistently detectable plasma EBV DNA

**DOI:** 10.18632/oncotarget.9323

**Published:** 2016-05-12

**Authors:** Wen-Yi Wang, Tian-Yun Lin, Chih-Wen Twu, Hsiao-Hui Tsou, Po-Ju Lin, Yi-Chun Liu, Jing-Wen Huang, He-Yuan Hsieh, Jin-Ching Lin

**Affiliations:** ^1^ Department of Nursing, Hung Kuang University, Taichung, Taiwan; ^2^ Department of Nursing, National Taichung University of Science and Technology, Taichung, Taiwan; ^3^ Department of Otorhinolaryngology, Taipei Veterans General Hospital, Taipei, Taiwan; ^4^ Department of Otorhinolaryngology, Taichung Veterans General Hospital, Taichung, Taiwan; ^5^ Faculty of Medicine, School of Medicine, National Yang-Ming University, Taipei, Taiwan; ^6^ Institute of Population Health Sciences, National Health Research Institutes, Miaoli, Taiwan; ^7^ Graduate Institute of Biostatistics, College of Public Health, China Medical University, Taichung, Taiwan; ^8^ Department of Radiation Oncology, Taichung Veterans General Hospital, Taichung, Taiwan; ^9^ Institute of Clinical Medicine, School of Medicine, National Yang Ming University, Taipei, Taiwan; ^10^ Department of Medicine, China Medical University, Taichung, Taiwan

**Keywords:** nasopharyngeal carcinoma, radiotherapy, plasma Epstein-Barr virus DNA, quantitative polymerase chain reaction

## Abstract

**Purpose:**

To investigate the long-term clinical outcome of nasopharyngeal carcinoma (NPC) patients with persistently detectable plasma EBV (pEBV) DNA after curative radiotherapy (RT).

**Results:**

The post-RT pEBV DNA levels were very lower copy number (median 21, interquartile range 8–206 copies/ml). After long-term follow-up, the relapse rate was 64.8%, the median time to progression 20 months, and 5-year overall survival (OS) 49.6%. Thirty-two of 39 (82.1%) patients with high viral load (≥ 100 copies/ml) developed tumor relapse, whereas 57.0% (49/86) patients with low viral load (< 100 copies/ml) had tumor relapse (*P* = 0.0065). The 5-year OS rates were 20.5% and 62.9% for patients with viral load ≥ and < 100 copies/ml (median survival, 20 vs. 100 months; *P* < 0.0001). Patients who received adjuvant chemotherapy (AdjCT) experienced significant reduction in distant failures (66.2% vs. 31.6%; *P* = 0.0001) but similar locoregional recurrences (*P* = 0.2337). The 5-year OS rates were 69.4% for patients who received AdjCT compared with 33.2% for those of without AdjCT (median survival, 111 vs. 32 months; *P* < 0.0001).

**Methods:**

We screened 931 newly diagnosed NPC patients who finished curative RT and found 125 patients (13.4%) with detectable pEBV DNA one week after RT. The clinical characteristics, treatment modality, subsequent failure patterns and survivals were analyzed.

**Conclusions:**

NPC patients with persistently detectable pEBV DNA after curative RT have a higher rate of treatment failure and poor survivals. Levels of the post-RT pEBV DNA and administration of AdjCT affect the final outcome significantly.

## INTRODUCTION

Nasopharyngeal carcinoma (NPC) has been demonstrated as a radio- and chemo-sensitive tumor. The current standard of care is radiotherapy (RT) alone for early-stage tumor and combined radio-chemotherapy for advanced-stage diseases [1–11]. Treatment failure in the past was due to a high rate of local recurrence and/or distant metastasis. However, advances in radiation oncology have improved the locoregional control, and treatment failure is now due mainly to distant metastasis.

Many clinicopathological variables were reported to predict treatment outcome. Among them, the TNM staging (extent of local invasion, regional lymphatic spread, and distant metastasis) is the most important prognostic factors in NPC. Recently, cell-free circulating EBV DNA can be detected in plasma and serum of NPC patients, and has been shown as a significant prognosticator at different time-points, including pre-treatment, post-treatment, and middle part of RT [12–22]. Our previous studies found that the post-treatment plasma EBV DNA (pEBV DNA) status (detectable or undetectable) dominated the effect of the pre-treatment viral load and had greater impact than the TNM staging [17–19]. Other investigators confirmed our findings [20, 21].

The long-term clinical behavior of NPC patients with persistently detectable pEBV DNA after curative RT with/without chemotherapy has rarely been reported. Thus, we investigate the clinical course in these high-risk patients after long-term follow-up and hope to serve as a reference for future trials of treatment strategy improvement.

## RESULTS

### Long-term treatment outcome for 125 patients with detectable pEBV DNA after RT

No patients were lost to follow. After a median follow-up of 99 months, there were 81 failures and 73 deaths until the time of this writing. The patterns of failures consisted of 9 in local, 6 in regional, 50 in distant, 3 in local + regional, 6 in local + distant, 5 in regional + distant, and 2 in local + regional + distant sites. The relapse rate was 64.8% and the median time to progression was 20 months. The 1-year, 3-year, and 5-year LRFFS rates were 93.4%, 79.3%, and 72.5%, respectively. The corresponding rates of DMFFS were 68.0%, 50.5%, and 49.6%, respectively. The 1-year, 3-year, and 5-year rates of OS were 93.6%, 60.0%, and 49.5%, respectively.

Patients without persisting pEBV DNA had a significantly lower relapse rate (172/806 = 21.3%) and better survivals (5-year LRFFS = 89.2%, 5-year DMFFS = 88.0%, and 5-year OS = 85.3%).

### Effects of the post-RT pEBV DNA viral load on subsequent relapses and survivals

**Figure 1:**
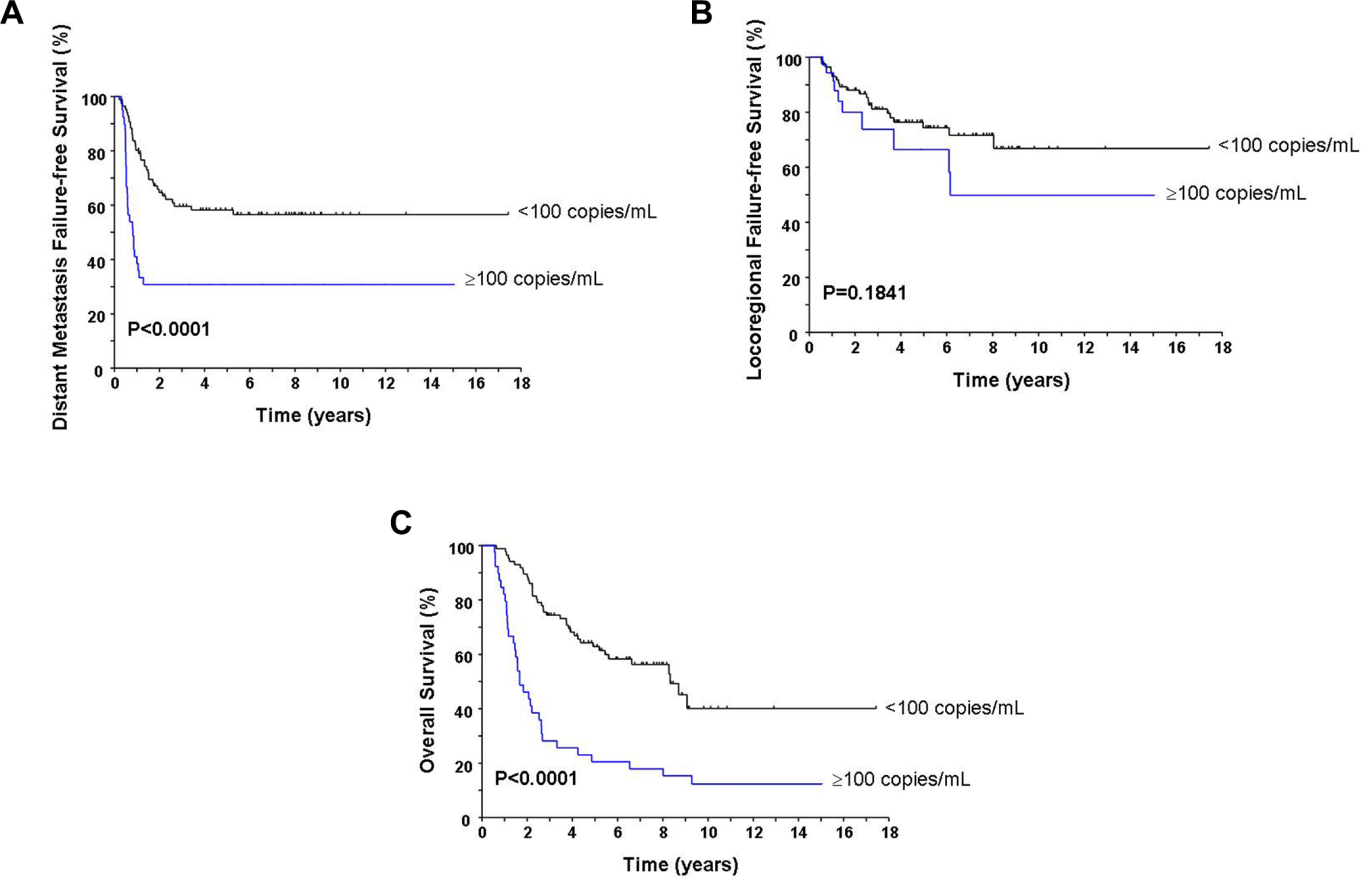
Comparison of the distant metastasis failure-free survival (A), locoregional failure-free survival (B), and overall survival (C) according to the post-RT pEBV DNA load

Table 1 lists the pre-treatment characteristics of the patients according to post-RT viral load. There were no statistically significant differences in terms of age, gender, pathologic type, T-classification, N-classification, overall stage, and modality of initial treatment between the patients with post-RT viral load ≥ and < 100 copies/mL. Thirty-two of 39 (82.1%) patients with post-RT pEBV DNA ≥ 100 copies/ml developed tumor relapse later, whereas 57.0% (49/86) patients with post-RT pEBV DNA < 100 copies/ml had tumor relapse (*P* = 0.0065). Patients with a lower viral load had significantly less distant failures (41.9% vs. 69.2%; *P* = 0.0046) but no difference in locoregional recurrences (24.4% vs. 25.6%; *P* = 0.8834). The 5-year DMFFS rates were 30.8% and 58.2% for the patients with post-RT viral load ≥ and < 100 copies/mL (*P* < 0.0001, Figure 1A). The 5-year LRFFS rates for the patients with high and low viral load were 66.5% and 74.4%, respectively (*P* = 0.1841, Figure 1B). The post-RT viral load affected OS significantly. The 5-year rates of OS were 20.5% and 62.9% for the patients with post-RT viral load ≥ and < 100 copies/mL (median survival, 20 vs. 100 months; HR, 0.22; 95% CI, 0.12 to 0.38; *P* < 0.0001, Figure 1C).

**Table 1:** Patient characteristics according to post-RT viral load (n = 125)

Characteristics	pEBV DNA levels				*P*
	≥ 100 copies/mL (*n* = 39)		< 100 copies/mL (*n* = 86)		
	No.	%	No.	%	
Median age, year	47		50		0.5911
95% CI	43–51		46–51		
Sex					0.8471
Male	27	69.2	61	70.9	
Female	12	30.8	25	29.1	
Pathology (WHO)					0.7611
Type I	0	0	1	1.2	
Type II	31	79.5	63	73.2	
Type III	8	20.5	22	25.6	
T-classification					0.4742
T1–2	19	48.7	36	41.9	
T3–4	20	51.3	50	58.1	
N-classification					0.1102
N0–2	20	51.3	57	66.3	
N3	19	48.7	29	33.7	
Overall stage					0.3656
II + III	13	33.3	36	41.9	
IV	26	66.7	50	58.1	
Previous treatment					0.4744
IndCT+RT	25	64.1	57	66.3	
CCRT	12	30.8	26	30.2	
IndCT+CCRT	2	5.1	1	1.2	
RT alone	0	0	2	2.3	

*Abbreviations:* IndCT = induction chemotherapy; RT = radiotherapy; CCRT = concurrent chemoradiotherapy; WHO = world health organization.

### Effects of the post-RT adjuvant chemotherapy on subsequent relapses and survivals

**Figure 2:**
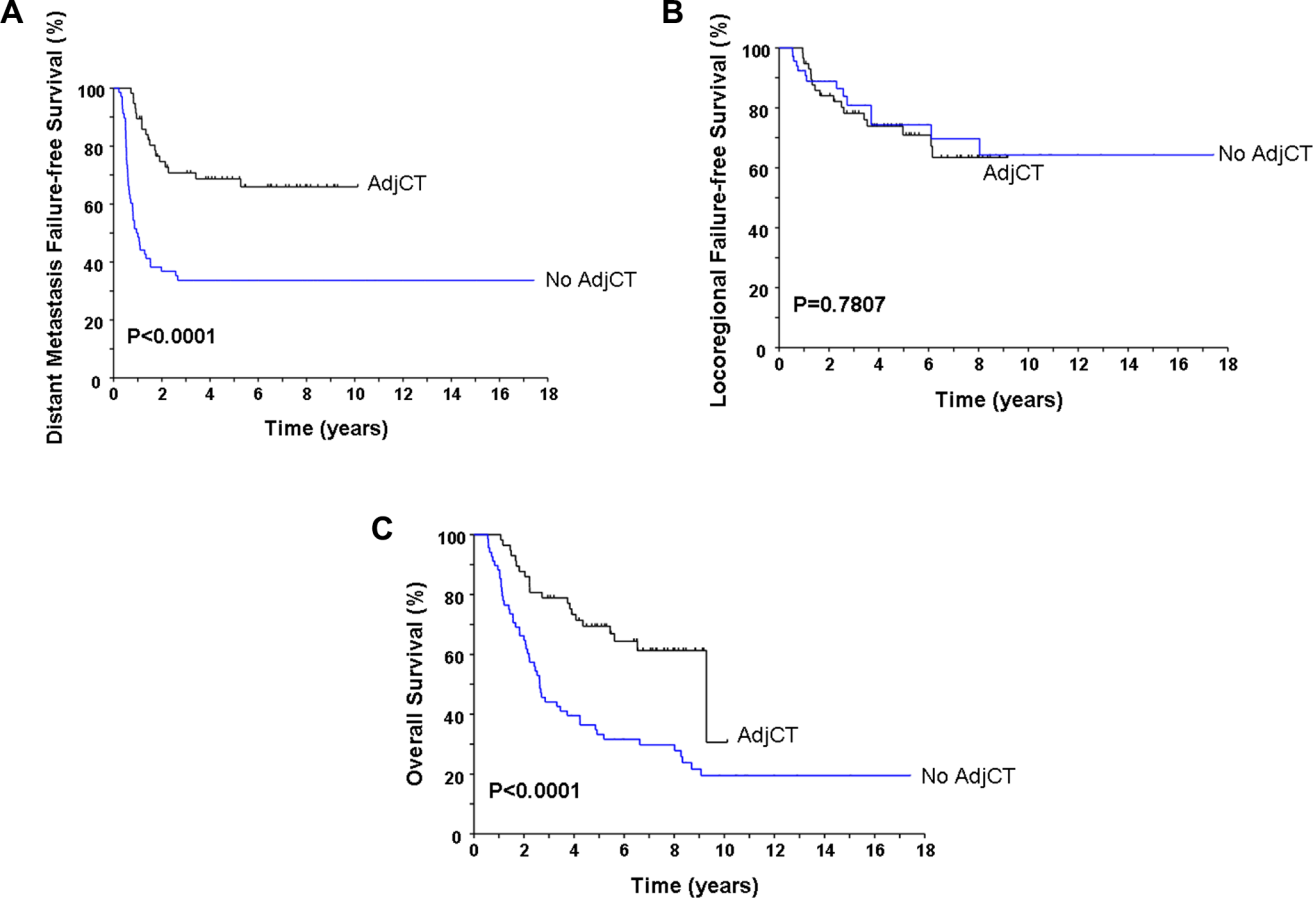
Comparison of the distant metastasis failure-free survival (A), locoregional failure-free survival (B), and overall survival (C) according to the delivery of adjuvant chemotherapy or not

Table 2 lists the pre-treatment characteristics of the patients according to AdjCT or not. No statistically significant differences in terms of age, gender, pathologic type, T-classification, N-classification, overall stage, and modality of initial treatment between the patients with and without AdjCT were observed. The relapse rates between the patients with and without AdjCT were 54.4% (31/57) and 73.5% (50/68), respectively (*P* = 0.0256). There were significant reduction in distant failures (31.6% vs. 66.2%; *P* = 0.001) but similar locoregional recurrences (29.8% vs. 20.6%; *P* = 0.2337) for patients who received AdjCT. The 5-year DMFFS rates for patients with and without AdjCT were 68.7% and 33.7% (*P* < 0.0001, Figure 2A), respectively. The 5-year LRFFS rates were 70.9% and 74.3% for the patients who received and did not receive AdjCT (*P* = 0.7807, Figure 2B). The benefits of AdjCT in the reduction of distant metastasis translated into affecting the OS. The 5-year OS rates were 69.4% for the patients who received AdjCT compared with 33.2% for those of without AdjCT (median survival, 111 vs. 32 months; HR, 0.38; 95% CI, 0.24 to 0.61; *P* < 0.0001, Figure 2C).

**Table 2:** Patient characteristics according to post-RT adjuvant chemotherapy (n = 125)

Characteristics	Adjuvant chemotherapy				*P*
	Yes (*n* = 57)		No (*n* = 68)		
	No.	%	No.	%	
Median age, year	48		49		0.7187
95% CI	45–51		45–52		
Sex					0.9598
Male	40	70.2	48	70.6	
Female	17	29.8	20	29.4	
Pathology (WHO)					0.3427
Type I	0	0	1	1.5	
Type II	46	80.7	48	70.6	
Type III	11	19.3	19	27.9	
T-classification					0.7393
T1–2	26	45.6	29	42.6	
T3–4	31	54.4	39	57.4	
N-classification					0.7430
N0–2	36	63.2	41	60.3	
N3	21	36.8	27	39.7	
Overall stage					0.8993
II + III	22	38.6	27	39.7	
IV	35	61.4	41	60.3	
Previous treatment					0.6605
IndCT+RT	37	64.9	45	66.2	
CCRT	18	31.6	20	29.4	
IndCT+CCRT	2	3.5	1	1.5	
RT alone	0	0	2	2.9	

*Abbreviations:* IndCT = induction chemotherapy; RT = radiotherapy; CCRT = concurrent chemoradiotherapy; WHO = world health organization.

Six of 39 patients with the high viral load and 51 of 86 patients with the low viral load received AdjCT. Table 3 summarized the survival impacts of AdjCT on both subgroups. AdjCT improved DMFFS (*P* = 0.0054) and OS (*P* = 0.0137) for patients with the low viral load subgroup. Although patients with AdjCT had better DMFFS (*P* = 0.0508) and OS (*P* = 0.3704) than those without AdjCT among the high viral load subgroup, the difference did not reach statistical significance.

**Table 3:** Effects of AdjCT in patients with high and low viral load after curative RT

Post-RT plasma EBV DNA	*n*	Overall survival		DMFFS		LRFFS	
		5-year rate (%)	*P*	5-year rate (%)	*P*	5-year rate (%)	*P*
High viral load (≥ 100 copies/ml)	39						
AdjCT (Yes)	6	50.0	0.3704	66.7	0.0508	66.7	0.2332
AdjCT (No)	33	15.2		24.2		66.1	
Low viral load (< 100 copies/ml)	86						
AdjCT (Yes)	51	71.6	0.0137	69.3	0.0054	71.5	0.8857
AdjCT (No)	35	50.6		42.5		78.8	

*Abbreviations:* AdjCT = adjuvant chemotherapy; RT = radiotherapy; DMFFS = distant metastasis failure-free survival; LRFFS = locoregional failure-free survival.

### Factors predicting local control

Both post-RT viral load (*P* = 0.6875) and AdjCT (*P* = 0.8391) had no significant effects on local failure-free survival for NPC patients with persistently detectable pEBV DNA after curative RT. Conventional T-classification affects local control significantly. The 5-year local failure-free survival rates were 93.4% for the T1-2 group compared with 74.5% for the T3-4 group (*P* = 0.0431). In addition, the initial gross target volume (GTV) of the primary tumor positively correlated with the T-classification also affected local control. The 5-year local failure-free survival rates for patients with initial GTV < vs. ≥ median value were 91.6% vs. 75.5% (*P* = 0.0541), respectively. Tumor response (complete response vs. non-complete response, *P* = 0.3540) evaluated 2–3 months after finishing RT showed no significant impact in predicting local control.

### Cox multivariate analysis

Variables with *P* values < 0.10 in the univariate analysis were incorporated in the multivariate Cox proportional hazards model. After controlling for confounding factors, the effects of post-RT viral load (HR, 0.37; 95% CI, 0.22 to 0.61; *P* < 0.001), AdjCT (HR, 0.44; 95% CI, 0.26 to 0.76; *P* = 0.0035), and overall stage (HR, 0.34; 95% CI, 0.16 to 0.69; *P* = 0.0029) on OS remained statistically significant. Similar results were observed in DMFFS analysis (*P* = 0.0171 for post-RT viral load, *P* = 0.0003 for AdjCT, and *P* = 0.0132 for overall stage). There were no significant independent factors in predicting LRFFS.

## DISCUSSION

NPC has been proven as an EBV-associated cancer for a long time. Recent development of the real-time quantitative polymerase chain reaction assay allows us to detect circulating EBV DNA fragments in most newly diagnosed NPC patients [12–22]. In addition, pEBV DNA was rarely detected in healthy people and long-term survivors of NPC patients without relapse [12, 17]. Many prior studies demonstrate that pEBV DNA can serve as a reliable biomarker in the detection, monitoring, and prognostic prediction for NPC [12–22].

Initially, pre-treatment circulating EBV DNA levels have been shown to correlate with the tumor load, clinical stage, and treatment outcome in NPC. Using a cutoff > or = 0 copies/mL, we report for the first time that pEBV DNA levels one week after finishing RT is the most important independent prognostic marker in predicting survivals [17]. In a later study with a minimal of 6 year follow- up and extending sample size, patients with persistently detectable post-RT pEBV DNA had significantly worse OS (HR = 4.61, 95% CI = 2.64–7.76, *P* < 0.001) and relapse-free survival (HR = 7.55, 95% CI = 4.35–13.12, *P* < 0.001) than those with undetectable pEBV DNA [18]. Moreover, the post-RT pEBV DNA status dominated the effect of any other variables, including the pre-treatment pEBV DNA load as well as the TNM staging. Other investigators adopted our cutoff value and showed similar outcome [20, 21]. Only one group from Hong Kong used a different cutoff value at post-RT pEBV DNA (> 500 copies/mL) that revealed significantly different effects on survival analysis for their patients [14]. However, the Hong Kong NPC Study Group adopted our cutoff value in analyzing 576 NPC patients enrolled in a prospective multicenter trial [23] and confirmed our results. They reported that the 3-year relapse-free survival (48.6% vs. 85.8%, *P* < 0.0001) and OS (69.9% vs. 94.5%, *P* < 0.0001) were significant worse for patients with post-RT detectable (> 0 copies/mL) than those with undetectable pEBV DNA after a median follow-up of 3.76 years. An ongoing large randomized trial by the Radiation Therapy Oncology Group (RTOG-1315 or NRG-HN001) selected the same cutoff value as ours (post-RT pEBV DNA > or = 0 copy/mL) to guide further treatment policy. Based on above discussion, we can identify a biomarker-selected high-risk subgroup of NPC patients with persistently detectable post-RT pEBV DNA. These patients need a more aggressive treatment strategy in future trials.

The current report supplements the data for these biomarker-selected high-risk patients after long-term follow-up. In addition, we further investigate the prognostic impacts of post-RT pEBV DNA concentrations and administration of AdjCT or not in these high-risk patients. Some clinically relevant findings are summarized below. First, only a few (13.4%) patients experienced persistently detectable pEBV DNA after RT with/without chemotherapy. Furthermore, the levels of post-RT pEBV DNA (median 21, interquartile range 8–206 copies/mL) for the 125 patients in this study were significantly lower compared with those of pre-treatment levels (median 1461, interquartile range 302–4390 copies/mL) for the 99 patients in our previous study [17]. These data support several established concepts that 1)NPC is a radio- and chemo-sensitive tumor, 2) the current treatment is highly effective for NPC, 3) most tumor cells are eradicated by the radio-chemotherapy, 4) the circulating EBV DNA of most patients drop from a high copy number to undetectable (0 copy/mL), and 5) only a few patients have detectable but in a very low copy number. Second, the prognostic impacts of the post-RT pEBV DNA levels among these high-risk patients have never been reported. Using an additional cutoff value of 100 copies/mL, this study showed that patients with a higher viral load (≥ 100 copies/mL) had a worse DMFFS and OS than those with a lower viral load (< 100 copies/mL). These findings provide us a new clinical implication that post-RT viral load should be considered as an important stratification factor or the design of different intensive adjuvant therapies for patients with a lower or higher viral load subgroup in future trials. Third, we confirmed our prior study results [24] that adding low-toxic metronomic AdjCT of oral tegafur-uracil with/without cyclophosphamide could reduce distant failure and improve overall survival in NPC patients with persistently detectable pEBV DNA after curative RT by increasing case numbers. Furthermore, subgroup analysis in this study revealed that AdjCT of oral agents benefited for patients with a low viral load but had inadequate benefits for patients with a higher viral load. A second new clinical implication of having another cutoff of 100 copies/mL is that adding more intensive adjuvant therapies (intravenous chemotherapeutic or targeted agents) before low-toxic metronomic oral agents should be tried for the high viral load subgroup. Prospective randomized trials are strongly recommended to investigate “what is the best regimen of AdjCT” for different subgroup patients.

The current NCCN guidelines recommend that CCRT using tri-weekly high dose cisplatin followed by AdjCT of PF regimen (cisplatin + 5-fluorouracil) for advanced (stage III–IV) NPC, originated from the results of the Intergroup study (2). However, reports from several previous meta-analyses revealed that no any benefit in using AdjCT for NPC patients [25–28]. These contradictions puzzle most oncologists for decades. A recent update of the meta-analysis actually shows that both CCRT alone and combined CCRT with AdjCT have survival benefits than RT alone [29]. In our opinion, routine delivery of post-RT AdjCT after RT (± induction/concurrent chemotherapy) for “all” advanced-stage NPC patients should be re-considered. Indeed, patients with advanced disease (stage III–IV) contained a heterogeneous group with variable relapse rate. Pre-treatment prognostic factors (patient's characteristics and initial clinical stages) are no longer important because the tumors in most patients are completely eradicated after initial definitive chemoradiotherapy. This might be one of the reasons why all previous pure AdjCT trial failed to prove its value. We propose that risk-grouping according to post-RT characteristics such as post-RT pEBV DNA load, not only according to pre-treatment factors is the key for future AdjCT trials. The ongoing randomized trial (NRG-HN001) by the RTOG has selected post-RT pEBV DNA levels (> or = copies/mL) as the critical-point for different treatment allocation. Patients with undetectable pEBV DNA after CCRT will be randomized to either 3 cycles AdjCT of PF or close observation. Whereas, patients with persistently detectable pEBV DNA (> 0 copies/mL) will be randomized to different AdjCT regimen (PF versus paclitaxel + gemcitabine). Taiwan Cooperative Oncology Group is also performing a phase III randomized trial to compare the outcome between immediate AdjCT (intravenous MEP followed oral tegafur-uracil) and delayed salvage chemotherapy in NPC patients with post-RT detectable pEBV DNA.

In summary, only 13.4% (125/931) patients have persistently detectable circulating EBV DNA but in very low copy number after RT with/without chemotherapy. These subgroup patients have a high subsequent relapse rate (64.8%) with predominantly distant failure. The median time to progression is only 20 months and the 5-year OS rate is 49.5%. Levels of the post-RT pEBV DNA and administration of AdjCT affect the final outcome. Future trials should consider post-RT pEBV DNA levels as a stratification factor and investigate the optimal AdjCT regimen for the target population.

## MATERIALS AND METHODS

The inclusion criteria for this retrospective study were patients with 1) previously untreated, biopsy-proven NPC, 2) no distant metastasis, 3) receiving curative RT with/without chemotherapy, and 4) persistently detectable pEBV DNA one week after RT. A total of 931 patients were screened and 125 patients (13.4%) with detectable pEBV DNA after RT were eligible for the final analysis. This study was approved by the Institutional Review Board of our hospital.

There were 88 males and 37 females. The median age was 48 years (range 20 to 82). By histology, 124 of 125 patients were identified as having nonkeratinizing carcinoma with differentiated (94 cases, WHO type IIa) or undifferentiated (30 cases, WHO type IIb) type. Only one patient was reported as keratinizing squamous cell carcinoma (WHO type I). The distribution of T-classification and N-classification were T1/T2/T3/T4 = 18/37/30/40 and N0/N1/N2/N3 = 2/18/57/48 patients. Most patients belong to overall stage IV (60.8%). The major modality of initial curative treatment were induction chemotherapy (IndCT) + RT (82 patients, 65.6%) and concurrent chemoradiotherapy (CCRT, 38 patients, 30.4%). Only 3 patients received IndCT + CCRT and 2 patient received RT alone.

Post-RT adjuvant chemotherapy (AdjCT) with oral tegafur-uracil (each capsule contained 100 mg tegafur and 224 mg uracil) 2 capsules twice daily for 12 months was administered for 57 patients. Among them, 24 patients also received oral cyclophosphamide 50 mg per day with tegafur-uracil and intravenous chemotherapy of MEP (mitomycin-C 8 mg/m^2^, epirubicin 60 mg/m^2^, and cisplatin 60 mg/m^2^, repeated every 3 weeks for 4–6 cycles) were delivered to 5 patients before tegafur-uracil.

Re-staging survey including computerized tomography (CT) scan or magnetic resonance imaging (MRI) from the skull base to the lower neck, chest X-ray, abdominal sonography and whole-body bone scan were performed 2–3 months after finishing RT. These image studies were repeated once every 6–12 months during the first 2 years, annually thereafter, and any time when recurrence/metastasis was suspected clinically. ^18^F-fluorodeoxyglucose positron emission tomography (PET) scan or PET/CT scan was not a routine work-up but was usually done if the above-mentioned examinations were equivocal or physicians in charge wanted to obtain a confirmatory diagnosis and evaluation of the extent of recurrence/metastasis before salvage treatment.

The frequencies of post-RT follow-up are every 1–2 months in the first year, every 2–3 months in the second and third years, every 3–6 months in the fourth and fifth years, and annually thereafter. Flexible nasopharyngoscopy was performed once per 3–6 months during the follow-up visits. All tumor recurrences/metastases were documented by imaging studies along with pathological verification if the lesions were accessible and patients agreed.

The primary end-points of this study were the patterns of failure and survivals in patients with detectable post-RT pEBV DNA. The secondary end-points were to investigate the prognostic impact of post-RT viral load and post-RT AdjCT on subsequent relapses and survivals. Analyzed outcomes included various relapse rates, time to progression, overall survival (OS), locoregional failure-free survival (LRFFS), and distant metastasis failure-free survival (DMFFS). OS defined as the duration from the first day of curative treatment to the date of death of any cause or the date of the last follow-up visit. LRFFS was calculated from the first day of curative treatment until the day of first occurrence of local, regional, or both locoregional relapse or until the date of the last follow-up visit. DMFFS was calculated from the first day of curative treatment until the day of distant relapse or the date of the last follow-up. Univariate comparison of survival curves were performed by use of the log-rank test. Cox proportional hazards model was used to estimate the hazard ratios (HR) and 95% confidence intervals (CI) by univariate and multivariate analyses. All analyses were two-sided and a *P* value of less than 0.05 was considered statistically significant. Analyses were performed by use of SAS (Version 8.0; SAS Institute, Inc., Cary, NC).
